# Supplementary material for: Understanding the light induced hydrophilicity of metal-oxide thin films
Source: Nat Commun. 2024 Jan 2;15:124. doi: 10.1038/s41467-023-44603-2 (PMC10761860; doi:10.1038/s41467-023-44603-2)
Supplement: Supplementary file 1 — Supplementary Information [file 41467_2023_44603_MOESM1_ESM.pdf]

Supplementary Information

Understanding the light induced hydrophilicity of metal-oxide thin films

Rucha Anil Deshpande[1,a], Jesper Navne[1,a], Mathias Vadmand Adelmark[1], Evgeniy Shkondin[1], Andrea Crovetto[1], Ole Hansen[1], Julien Bachmann[1,2], and Rafael Taboryski[1,*]

[1] Technical University of Denmark, DTU Nanolab, National Centre for Nano Fabrication and Characterization, Ørsteds Plads B347, DK-2800 Kgs. Lyngby, Denmark

[2] Friedrich-Alexander-Universität Erlangen-Nürnberg, Chemistry of Thin Film Materials, IZNF, Cauerstr. 3, 91058 Erlangen, Germany

[a] These authors contributed equally.

[*] Corresponding author: rata@dtu.dk

Supplementary note

In the derivation of our model we assume no surface roughness dependence on layer thickness d. However, quantities such as the area density of surface states scale with inverse proportion to the effective surface area. To check this dependence, we characterized the surfaces by atomic force microscopy (AFM) (see Supplementary Figure 1). The results revealed that for ZnO, the effective surface area slightly did depend on layer thickness, however only by less than 4 % for TiO_2 and by less than 6 % for ZnO (Supplementary Figure 1), which is somewhat lower than the fitting uncertainties in Figure 5. Furthermore, surface roughness also has an impact on the transmission coefficients of the interface between air and the MOx. Roughness on subwavelength scale is known to have an antireflective effect.[1] However, the relatively low surface roughness encountered on the fabricated samples is expected to reduce the surface reflectance only by ~5%, which is insignificant compared to other uncertainties in our model.

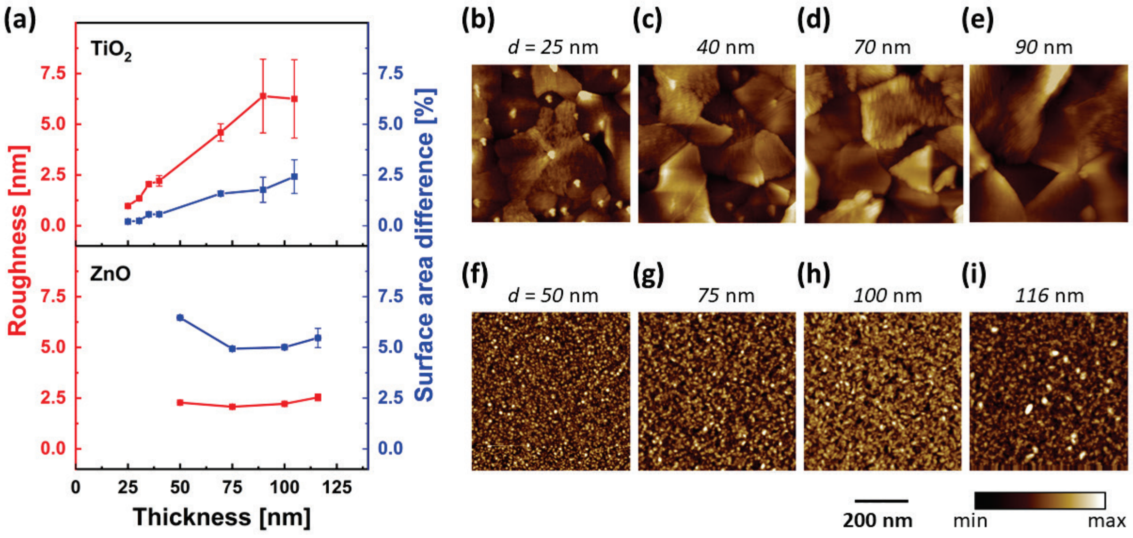

Supplementary Figure 1: MOx surface morphology imaged by AFM. (a) Roughness (root mean square of vertical variations across sample area) and surface area difference for different thicknesses of both TiO_2 and ZnO materials deposited by ALD on Si wafers. Vertical error bars represent one STD obtained from the AFM data analysis software. (b-e) AFM images of TiO2 layer thicknesses 25, 40, 70, 90 nm, respectively. (f-i) Images of ZnO layer thicknesses 50, 75, 100, 116 nm respectively.

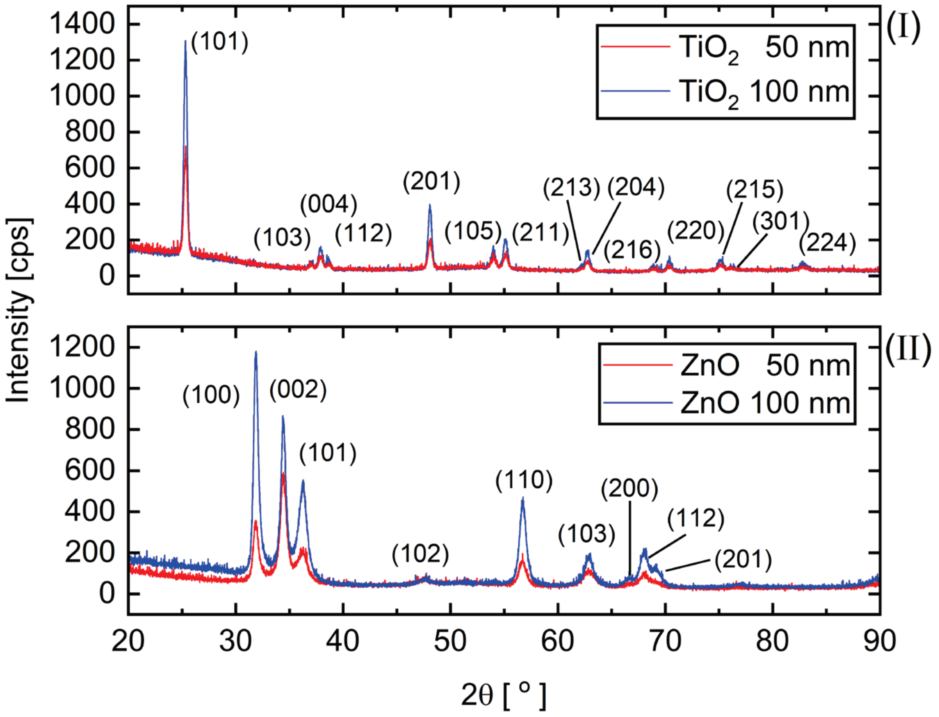

Supplementary Figure 2: Grazing-Incident X-ray Diffraction (GI-XRD) scans for two TiO_2 films (I) and two ZnO films (II). All peaks in the TiO_2 films can be attributed to anatase TiO_2 (see, e.g., collection code 9852 in the Inorganic Crystal Structure Database[2]. Peak widths and film texture are approximately thickness independent. The peak intensities tend to double when the thickness is doubled, indicating that the fraction of crystallized material within the film does not significantly change with film thickness.

Supplementary derivation

For an incident wave propagating in the positive z-direction $E_0 e^{ik_1 z}$=$E_0 e^{\frac{i2\tilde{n}_1 \pi z}{\lambda}}$ with wavelength λ (in vacuum/air), and amplitude E_0 we get the following infinite sum of roundtrips that contribute to the reflected wave.

$$E_{tot,r}(z) = \left(r_{12} + t_{12} r_{23} t_d^2 t_{21} \sum_{j=0}^{\infty} \left(r_{23} t_d^2 r_{21} \right)^j \right) E_0 e^{\frac{-i2\pi\tilde{n}_1 z}{\lambda}} = \left(r_{12} + \frac{t_{12} r_{23} t_d^2 t_{21}}{1 - r_{23} t_d^2 r_{21}} \right) E_0 e^{\frac{-i2\pi\tilde{n}_1 z}{\lambda}},$$

where we have exploited a mathematical identity for infinite series, and where $t_d = e^{\frac{i2\pi\tilde{n}_2 d}{\lambda}}$ is the phase factor picked up from traversal of the MOx layer. Likewise, we get for the transmitted wave that travel into the bulk of the Si.

$$E_{tot,t}(z) = \left(t_{12} t_{23} t_d \sum_{j=0}^{\infty} \left(r_{23} t_d^2 r_{21} \right)^j \right) E_0 e^{\frac{i2\pi\tilde{n}_1 z}{\lambda}} = \left(\frac{t_{12} t_{23} t_d}{1 - r_{23} t_d^2 r_{21}} \right) E_0 e^{\frac{i2\pi\tilde{n}_1 z}{\lambda}}.$$

The total transmittance and reflectance are then given by

$$R(d) = \left\| E_{tot,r}(z)/E_0 \right\|^2 \text{and } T(d) = \left\| E_{tot,t}(z)/E_0 \right\|^2$$

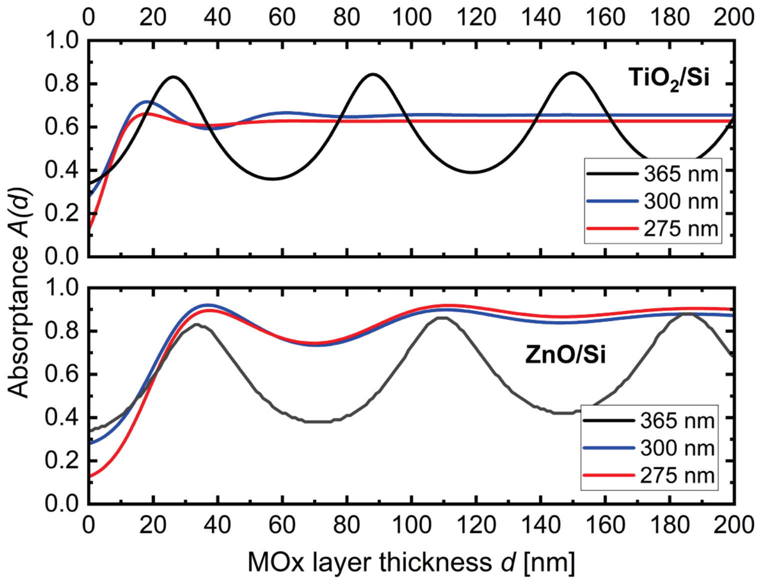

Supplementary Figure 3: Computed absorptance as a function of MOx layer thickness as $A(d) = 1 - R(d) - T(d)$ for TiO2/Si and Zn/Si heterojunctions for the wavelengths 365, 300 and 275 nm. In the computations, the measured Fresnel coefficients $\tilde{n}_j = n_j + i\kappa_j$ were used to obtain $R(d)$ and $T(d)$ by using Equation 10 and Equation 11.

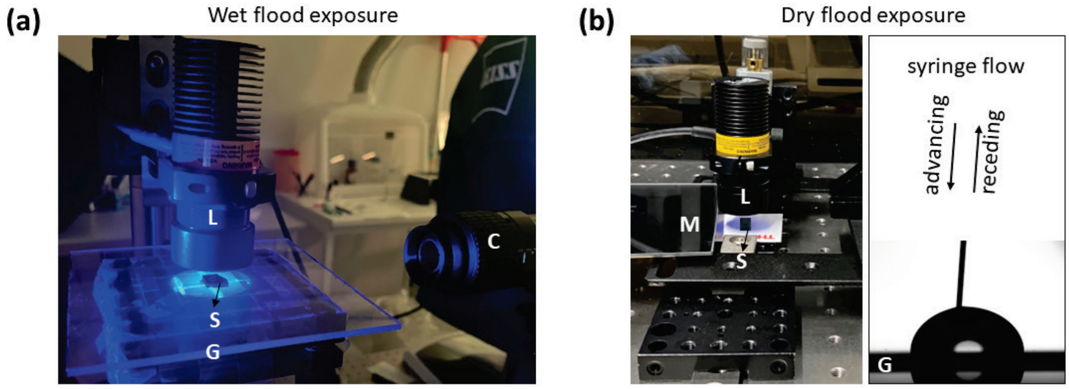

Supplementary Figure 4: Experimental set-up for photo-induced switching. Abbreviations L-UV LED lamp, S-sample, G-goniometer stage, C-camera, M-Mask (a) Wet exposure allows water droplet to be simultaneously measured with UV light exposure. The water droplet is pipetted out and UV light is exposed from top as shown in the image. (b) Dry exposure allows UV exposure on plain samples at different exposure times and its effect on the wettability switching is subsequently measured on the goniometer with advancing and receding contact angles. The image shows a sample exposed with UV light, with a mask holder attachment and its measurement made in goniometer. The mask allows for selective area exposure.

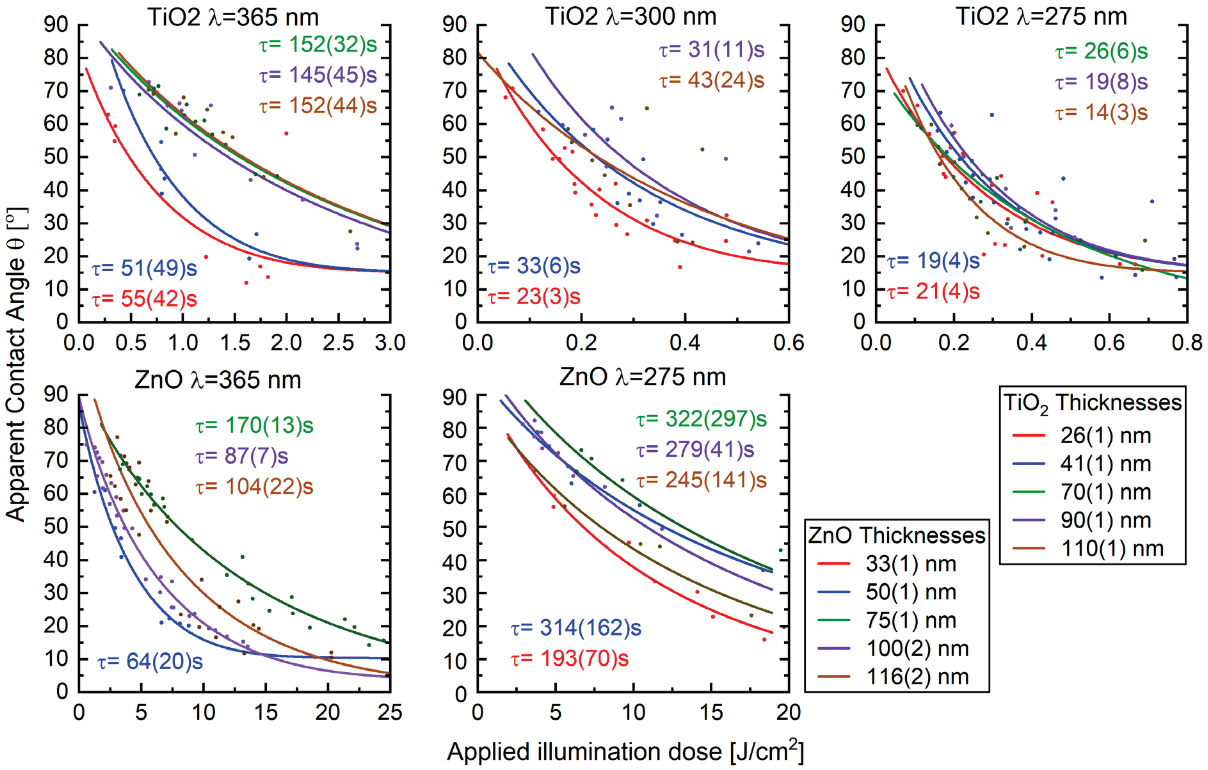

Supplementary Figure 5: Display of the model fit (line) to Equation 8 on photo-switches (dots) from multiple measurements. The figure shows a selection of thicknesses investigated. The time of the experiments has been converted to doses by multiplying the power with time and dividing with the lamp's spot size for easier comparison of the efficiency of the photo-induced hydrophilicity.

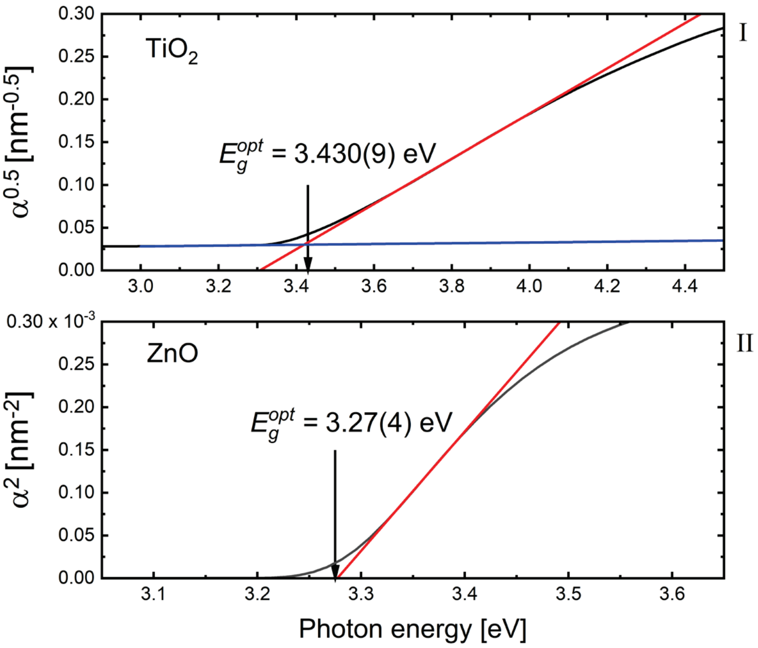

Supplementary Figure 6: Determination of optical bandgaps, I, TiO_2 and II, ZnO. The absorption coefficient α is given by the extinction coefficient κ (see Figure 3a) and the illumination wavelength λ as $\alpha = 4\pi/\lambda$. For predominantly crystalline semiconductors with indirect bandgap, such as anatase TiO_2, the bandgap is estimated from a plot of $\sqrt{\alpha}$ vs the photon energy ($h\upsilon$) as the extrapolation of the linear region to the line representing the background absorption as shown. This results in an estimate of the optical bandgap for TiO_2 of $E_g^{opt} = 3.430(9)$ eV. For TiO_2, the method is however prone to error from the choice of the background subtraction line. In the fit, the background line is allowed to have a slope. If a zero slope fit is used, we get $E_g^{opt} = 3.39(5)$ eV. A fair determination with a conservative error estimation is thus $E_g^{opt} = 3.43(2)$ eV for TiO_2. For ZnO, the same procedure is applied, except that here, the linear region of α^2 is extrapolated to the zero absorption point as no background subtraction is required. This yields $E_g^{opt} = 3.27(4)$ eV for ZnO. The error estimation is based on the errors propagated from the linear fits, where the fitting procedure yielding the slopes and axis intersections of the straight lines also yields the errors on slopes and axis intersections.

Supplementary References

1 Schneider, L., Feidenhans'l, N. A., Telecka, A. & Taboryski, R. J. One-step Maskless Fabrication and Optical Characterization of Silicon Surfaces with Antireflective Properties and a White Color Appearance. *Scientific Reports* **6** (2016). https://doi.org/10.1038/srep35183

2 Zagorac, D., Mueller, H., Ruehl, S., Zagorac, J. & Rehme, S. Recent developments in the Inorganic Crystal Structure Database: theoretical crystal structure data and related features. *Journal of Applied Crystallography* **52**, 918-925 (2019). https://doi.org/10.1107/s160057671900997x
